# Metronomic gemcitabine suppresses tumour growth, improves perfusion, and reduces hypoxia in human pancreatic ductal adenocarcinoma

**DOI:** 10.1038/sj.bjc.6605727

**Published:** 2010-06-08

**Authors:** K K Y Cham, J H E Baker, K S Takhar, J A Flexman, M Q Wong, D A Owen, A Yung, P Kozlowski, S A Reinsberg, E M Chu, C-W A Chang, A K Buczkowski, S W Chung, C H Scudamore, A I Minchinton, D T T Yapp, S S W Ng

**Affiliations:** 1 Department of Advanced Therapeutics, British Columbia Cancer Agency, 675 West 10th Avenue, Vancouver, British Columbia, Canada V5Z 1L3; 2 Department of Medical Biophysics, British Columbia Cancer Agency, Vancouver, British Columbia, Canada; 3 Faculty of Pharmaceutical Sciences, University of British Columbia, Vancouver, British Columbia, Canada; 4 Department of Pathology & Laboratory Medicine, Faculty of Medicine, University of British Columbia, Vancouver, British Columbia, Canada; 5 Magnetic Resonance Imaging Research Centre, University of British Columbia, Vancouver, British Columbia, Canada; 6 Department of Surgery, Faculty of Medicine, University of British Columbia, Vancouver, British Columbia, Canada

**Keywords:** tumour microenvironment, pancreatic cancer, metronomic chemotherapy, gemcitabine, anti-angiogenesis

## Abstract

**Background:**

The current standard of care for pancreatic cancer is weekly gemcitabine administered for 3 of 4 weeks with a 1-week break between treatment cycles. Maximum tolerated dose (MTD)-driven regimens as such are often associated with toxicities. Recent studies demonstrated that frequent dosing of chemotherapeutic drugs at relatively lower doses in metronomic regimens also confers anti-tumour activity but with fewer side effects.

**Methods:**

Herein, we evaluated the anti-tumour efficacy of metronomic *vs* MTD gemcitabine, and investigated their effects on the tumour microenvironment in two human pancreatic cancer xenografts established from two different patients.

**Results:**

Metronomic and MTD gemcitabine significantly reduced tumour volume in both xenografts. However, *K*^trans^ values were higher in metronomic gemcitabine-treated tumours than in their MTD-treated counterparts, suggesting better tissue perfusion in the former. These data were further supported by tumour-mapping studies showing prominent decreases in hypoxia after metronomic gemcitabine treatment. Metronomic gemcitabine also significantly increased apoptosis in cancer-associated fibroblasts and induced greater reductions in the tumour levels of multiple pro-angiogenic factors, including EGF, IL-1*α*, IL-8, ICAM-1, and VCAM-1.

**Conclusion:**

Metronomic dosing of gemcitabine is active in pancreatic cancer and is accompanied by pronounced changes in the tumour microenvironment.

Pancreatic ductal adenocarcinoma remains a therapeutic challenge in oncology. The 5-year survival rate for unresectable disease is <5% and the median survival is 4–6 months. Current standard treatment consists of weekly gemcitabine administered at 1000 mg m^−2^ for 3 weeks, followed by a week of rest before the next cycle begins. Myelosuppression is the dose-limiting toxicity. This pulsatile maximum tolerated dose (MTD)-driven gemcitabine regimen significantly improves clinical benefit response but only provides modest survival benefits (Burris *et al*, 1997). Previous studies indicated that the efficacy and toxicity of gemcitabine are strongly schedule dependent (Hertel *et al*, 1990; Braakhuis *et al*, 1991, 1995; Boven *et al*, 1993; Veerman *et al*, 1996). In various murine and human tumour models, 120 mg kg^−1^ of gemcitabine administered every 3 days was shown to induce greater tumour growth inhibition than 2.5–3.5 mg kg^−1^ of gemcitabine injected daily or 240 mg kg^−1^ of the drug given weekly (Braakhuis *et al*, 1991, 1995). Furthermore, daily administration of gemcitabine at 1 mg kg^−1^ was reported to be more toxic than once-weekly administration at 40 mg kg^−1^ in one study (Hertel *et al*, 1990), but was demonstrated to be non-toxic in another (Laquente *et al*, 2008). Although these observations illustrated the importance of dose scheduling in maximising the anti-tumour efficacy and minimising the toxicity of gemcitabine, the physiological changes induced by different gemcitabine treatment schedules in the whole pancreatic tumour *in situ* have not been investigated. Understanding the effect of schedule-dependent drug dosing on tumour physiology will likely lead to the development of more efficacious gemcitabine monotherapy or combination chemotherapy with molecularly targeted agents for pancreatic cancer.

The therapeutic concept of administering cytotoxic agents continuously at lower doses relative to MTDs without drug-free breaks over extended periods, known as ‘metronomic chemotherapy’, is increasingly being recognised as a viable cancer treatment option. In comparison with MTD regimens, metronomic dosing protocols demonstrated reduced toxicity but similar or even increased efficacy in pre-clinical studies (Browder *et al*, 2000; Bertolini *et al*, 2003). More importantly, several Phase II trials have now shown that metronomic chemotherapy regimens using oral cyclophosphamide, methotrexate, etoposide, or capecitabine, in the absence or presence of agents with anti-angiogenic properties (e.g., thalidomide, celecoxib, bevacizumab), are active and minimally toxic in advanced breast cancer (Colleoni *et al*, 2006; Dellapasqua *et al*, 2008), recurrent ovarian cancer (Garcia *et al*, 2008), hormone refractory prostate cancer (Lord *et al*, 2007), and non-Hodgkin's lymphoma (Buckstein *et al*, 2006) among other cancers (Kieran *et al*, 2005; Young *et al*, 2006). The mechanistic basis of metronomic chemotherapy is believed to be primarily anti-angiogenic, either by direct killing of endothelial cells in the tumour vasculature (Browder *et al*, 2000; Klement *et al*, 2000), destruction of bone marrow-derived endothelial progenitors (Bertolini *et al*, 2003; Shaked *et al*, 2005), and/or stimulation of the release of endogenous anti-angiogenic factors such as thrombospondin-1 from perivascular stromal cells (Hamano *et al*, 2004). Moreover, immunostimulation has also been shown to mediate the anti-tumour effects of metronomic cyclophosphamide (Hermans *et al*, 2003; Ghiringhelli *et al*, 2004; Loeffler *et al*, 2005). As the majority of pancreatic cancer patients are elderly and often have poor performance status, metronomic gemcitabine regimens could be advantageous compared with conventional MTD-driven weekly gemcitabine, provided that it is better tolerated and equally or more efficacious. In this study, we sought to determine whether gemcitabine administered at lower doses in a metronomic regimen has anti-tumour activity in pancreatic ductal adenocarcinoma. The first objective was to evaluate the anti-tumour efficacy of low-dose metronomic gemcitabine *vs* MTD-driven weekly gemcitabine, and the second was to examine their effects on the tumour microenvironment in primary pancreatic cancer xenografts grown orthotopically in the pancreas of SCID mice.

## Materials and methods

### Establishment of orthotopic primary pancreatic cancer xenografts

Fresh pancreatic ductal adenocarcinoma tissues were obtained, with institutional ethics review board approval, from patients undergoing Whipple resection for pancreatic cancer at Vancouver General Hospital between 2006 and 2008. Resected, non-diagnostic specimens were washed twice in antibiotic-containing RPMI 1640. Necrotic tissues were removed, and viable tumour tissues were cut into small pieces of ∼2 × 2 × 2 mm in size and implanted subcutaneously into male C.B-17 SCID mice (Taconic, Germantown, NY, USA). When the subcutaneous tumours reached 600–800 mm^3^, they were excised and cut into small pieces of ∼2 × 2 × 2 mm in size for surgical implantation into the pancreas of groups of SCID mice as previously described (Ng *et al*, 2001). Two primary pancreatic ductal adenocarcinoma xenograft lines (PaCa8 and PaCa13) established from resected tumour tissues of two different patients were used in this study. All animal studies were conducted in accordance with the guidelines of the Canadian Council for Animal Care.

### Treatment regimens

Gemcitabine hydrochloride (Gemzar; Eli Lilly Canada Inc., Toronto, ON, Canada) was purchased from the British Columbia Cancer Agency pharmacy and dissolved in 0.9% saline (Hospira Healthcare Corp., Montreal, QC, Canada). When the orthotopic xenografts reached a palpable size of ∼200–400 mm^3^, tumour-bearing mice were randomised into three groups (*n*=5 each) and treated with vehicle control (0.9% saline, i.p.), one cycle of MTD gemcitabine (240 mg kg^−1^ on days 1, 8, and 15, i.p., followed by a 1-week break), or metronomic gemcitabine (30 mg kg^−1^ on days 1, 4, 7, 10, 13, 16, 19, 22, 25, and 28, i.p.) for 4 weeks. The dose and schedule used in the MTD gemcitabine regimen were chosen to emulate clinical treatment protocols. When administered on a weekly basis, 240 mg kg^−1^ (∼724 mg m^−2^ for a 20-g mouse) was previously reported to be the MTD of gemcitabine in mice (Veerman *et al*, 1996). Animal weight was monitored twice a week during the course of treatment.

### Dynamic contrast-enhanced magnetic resonance imaging

All studies were conducted using a 7.0 T magnetic resonance scanner (Bruker, Ettlingen, Germany), with a quadrature birdcage coil for transmission and a 1.7 × 1.4 cm rectangular surface coil for reception. At the end of the treatment cycle (day 29), the three groups of tumour-bearing mice were anaesthetised with isoflurane (Baxter Corporation, Mississauga, ON, Canada) and administered an i.v. bolus injection of the magnetic resonance contrast agent gadolinium (Gd)-DTPA (Omniscan; Nycomed, Oslo, Norway) at 0.3 mmol kg^−1^. Serial images were acquired to monitor changes in the concentration of the contrast agent in each pixel during the initial uptake and subsequent washout in the tumour. Magnetic resonance imaging scans followed the technique previously described by Lyng *et al* (1998). Briefly, a proton-density-weighted spin-echo MSME scan (TE/TR=11.9/6000 ms) was first acquired to serve as a baseline for conversion of pixel intensity to absolute concentration values of the contrast agent. Gd-DTPA was injected using a tail-vein catheter over ∼15 s. Starting at the time of injection, a series of 41 consecutive T_1_-weighted scans were acquired, with each scan lasting 64 s (spin-echo MSME, TR/TE=11.9/500 ms). In-plane spatial resolution was 312 *μ*m with 10–14 slices at a thickness of 1 mm or 1.5 mm, depending on the size of the tumour. The Gd-DTPA concentration–time curve for each pixel was fitted to a two-compartment Kety model (Kety, 1960; Tofts *et al*, 1999) that describes the pharmacokinetics of the contrast agent using two parameters: *K*^trans^, volume transfer constant between vascular space and extravascular extracellular space; and *v*_e_, fractional volume of extravascular extracellular space. *K*^trans^, which is often a mixed measure of blood flow and vascular permeability (Leach *et al*, 2003), was determined using in-house software and expressed as median values of *K*^trans^ in viable tumour tissues.

### Immunofluorescence staining and determination of tumour hypoxia, cell proliferation, and microvessel density

At the end of the treatment cycle (day 29), the three groups of tumour-bearing mice were injected with hypoxia marker EF5 (Dr Cameron Koch, University of Pennsylvania, PA, USA) at 30 mg kg^−1^ i.v. and with proliferation marker BrdU (Sigma, St Louis, MO, USA) at 1500 mg kg^−1^ i.p. at 3 h and 2 h before euthanasia, respectively. The orthotopic tumours were excised immediately after euthanasia. Perpendicular tumour diameters were measured with a caliper, and tumour volumes were calculated using the formula *π*/6 × *a* × *b*^2^, where *a* is the longest dimension of the tumour and *b* is the width. Tumours were then snap frozen in OCT (Sakura Finetek, Torrance, CA, USA) in liquid nitrogen. Cryosections of 10 *μ*m were cut from each tumour and fixed in 50% (v/v) acetone/methanol for 10 min at room temperature. Endothelial cells were stained with a monoclonal rat anti-mouse CD31/PECAM-1 antibody (1 : 100; BD PharMingen, San Diego, CA, USA), followed by an Alexa 647-conjugated goat anti-rat secondary antibody (1 : 200; Molecular Probes, Eugene, OR, USA). Reduced EF5 adducts in viable hypoxic cells were stained on the same section using a Cy3-conjugated monoclonal antibody to ELK3-51 (1 : 200) (Lord *et al*, 1993). Staining for incorporated BrdU was performed as previously reported (Kyle *et al*, 2003). Sections were counterstained with haematoxylin, dehydrated, and mounted. At each stage of staining, whole tumour sections were imaged ( × 10 objective) using a robotic fluorescence microscope (Zeiss Imager Z1, Oberkochen, Germany), a cooled monochrome CCD camera (Retiga 4000R, QImaging, Surrey, BC, Canada), a motorised slide loader and x-y stage (Ludl Electronic Products, Hawthorne, NY, USA), and customised NIH ImageJ software (http://rsb.info.nih.gov/ij/) running on a G5 Macintosh computer (Apple, Cupertino, CA, USA). All parameters stained on the same section were imaged separately, and subsequently overlaid and aligned with the removal of imaging artefacts. Tumour hypoxia and cell proliferation were visualised by EF5+ and BrdU+ staining, respectively. False colour images were generated using Adobe Photoshop CS3. Using NIH software applications and user-supplied algorithms, images were cropped to tumour tissue boundaries with necrosis removed; fluorescent images were inverted and a threshold was applied to the CD31 image. Neighbouring CD31+ pixels were grouped as CD31+ objects (i.e., vessels). All image pixels were sorted to determine their distance from the nearest vessels and the average distance between vessels, which reflects microvessel density, was reported. A short average distance between vessels indicates high microvessel density. The extent of tumour hypoxia or cell proliferation was reflected by the percentage of EF5+ or BrdU+ pixels, respectively, which was calculated as the number of EF5+ or BrdU+ pixels divided by the total number of tumour tissue pixels, and multiplied by 100%.

### Assessment of stromal cell apoptosis in primary xenografts

Cryosections of 10 *μ*m were cut, air dried, and fixed in 50% (v/v) acetone/methanol for 10 min at room temperature. Sections were stained with H&E for histological examination. To detect apoptosis in cancer-associated fibroblasts, sections were double stained with a FITC-conjugated monoclonal anti-*α*SMA antibody (1 : 200; Sigma) and an anti-cleaved-caspase 3 antibody (1 : 200; Cell Signaling Technology, Danvers, MA, USA), followed by an Alexa 546-conjugated secondary antibody (1 : 200; Molecular Probes). To determine apoptosis in endothelial cells, sections were incubated with anti-CD31/PECAM-1 antibody (1 : 100), followed by a FITC-conjugated secondary antibody (1 : 200; Jackson ImmunoResearch Laboratories, West Grove, PA, USA), and with anti-cleaved-caspase 3 antibody, followed by the Alexa 546-conjugated secondary antibody (1 : 200). Images of whole tumour sections were captured ( × 20 objective) using a Leica fluorescence microscopic imaging system (Leica Microsystems Inc, Richmond Hill, ON, Canada) equipped with both cooled 350FX monochrome and 300FX colour cameras for fluorescence and bright field imaging, respectively, an automated scanning stage (DM6000B), and Surveyor software (Objective Imaging, Kansasville, WI, USA). After removal of artefacts, composite tumour images for *α*SMA, CD31, and cleaved-caspase 3 staining were overlaid, aligned, thresholded, and analysed using in-house software programs developed in MATLAB (The Mathworks, Natick, MA, USA). The percentage of apoptotic cancer-associated fibroblasts (CAFs) or endothelial cells was reported as the number of *α*SMA+cleaved-caspase 3+ or CD31+cleaved-caspase 3+ pixels divided by the total number of *α*SMA+ or CD31+ pixels, respectively, and multiplied by 100%.

### Evaluation of tumour levels of growth factors and cytokines

Harvested tumours from all treatment groups were homogenised in lysis buffer (50 mM HEPES, 10% glycerol, 1% Triton X-100, 150 mM NaCl, 1 mM EDTA, 1.5 mM MgCl_2_, 100 mM NaF, 10 mM Na_4_P_2_O_7_, 100 *μ*g ml^−1^ PMSF, 5 *μ*g ml^−1^ leupeptin, and 5 *μ*g ml^−1^ aprotinin) using a Polytron PT10-35 homogeniser (Kinematica AG, Lucerne, Switzerland) and stored at −80°C. Total protein concentration in each tumour lysate was determined using a Micro BCA proteins assay kit (Pierce, Rockford, IL, USA). The protein levels of 89 human antigens that include numerous growth factors and cytokines were quantified in the tumour lysates using HumanMAP Antigen version 1.6 (Rules Based Medicine, Austin, TX, USA).

### Statistics

All results were presented as mean±s.e. Comparisons were made with one-way analysis of variance, followed by the Newman–Keuls test or the Dunnett test, with *P*<0.05 as the criterion for statistical significance.

## Results

### Anti-tumour activity of metronomic gemcitabine

**Figure 1:**
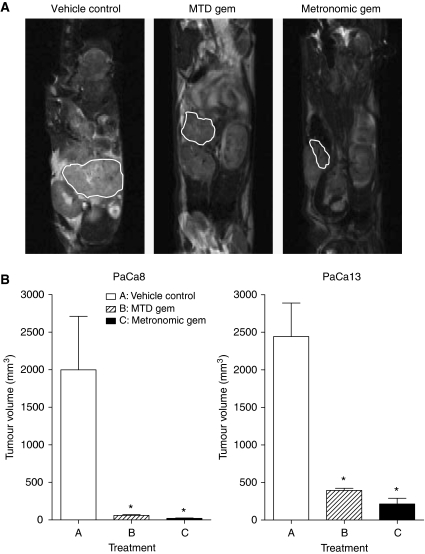
(**A**) Representative anatomical magnetic resonance scans of PaCa13 tumours after treatment with vehicle control (0.9% saline), maximum tolerated dose gemcitabine (MTD gem; 240 mg kg^−1^ on days 1, 8, and 15), or metronomic gemcitabine (30 mg kg^−1^, q3d) for 4 weeks. (**B**) Effects of different treatments on PaCa8 and PaCa13 tumour growth. Columns, mean of 4–5 tumours; bars, s.e. ^*^P<0.05, significantly different from that of vehicle control.

Representative anatomical magnetic resonance scans of PaCa13 tumours after treatment are shown in Figure 1A. The effects of treatments on tumour volume in the two primary tumour xenograft lines are summarised in Figure 1B. Metronomic and MTD gemcitabine significantly decreased the volume of PaCa8 tumours by 99 and 97%, respectively, and that of PaCa13 tumours by 91 and 84%, respectively, compared with vehicle control. There was a trend towards greater tumour volume reductions in the metronomic gemcitabine-treated group compared with the MTD gemcitabine-treated group. Neither of the treatments caused any significant weight loss in tumour-bearing mice relative to the vehicle control, indicating a lack of toxicity (data not shown).

### Morphological changes induced by metronomic gemcitabine in primary pancreatic cancer xenografts

**Figure 2:**
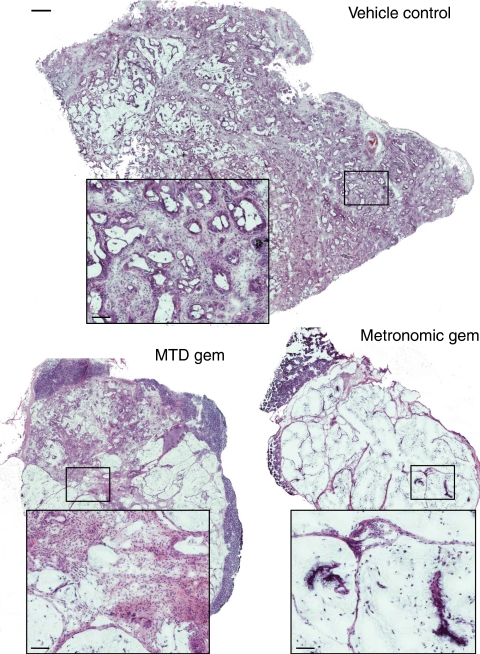
Representative composite haematoxylin and eosin (H&E) images (scale bar: 500 *μ*m) of PaCa8 tumours after treatment with vehicle control (0.9% saline), maximum tolerated dose gemcitabine (MTD gem; 240 mg kg^−1^ on days 1, 8, and 15), or metronomic gemcitabine (30 mg kg^−1^, q3d) for 4 weeks. *Squares*, magnified images (scale bar: 83 *μ*m) of the corresponding areas in the tumour sections.

H&E staining of vehicle control-treated PaCa8 xenografts showed that they are moderately differentiated ductal adenocarcinomas (Figure 2), whereas PaCa13 xenografts are poorly differentiated ductal adenocarcinoma (data not shown). PaCa8 tumours were debulked and seemed to have undergone mucinous differentiation, with few viable cancer cells remaining after metronomic gemcitabine treatment. Mucinous differentiation was also evident, although to a much lesser extent, with more viable cancer cells remaining in MTD gemcitabine-treated PaCa8 tumours (Figure 2). Similar morphological changes, but of lesser extent, were noted in PaCa13 tumours after metronomic and MTD gemcitabine treatment, with the former inducing more pronounced effects than the latter (data not shown). Overall, metronomic gemcitabine exerted greater histological effect than MTD gemcitabine in the two xenografts.

### Effects of metronomic gemcitabine on *K*^trans^ in primary xenografts

**Figure 3:**
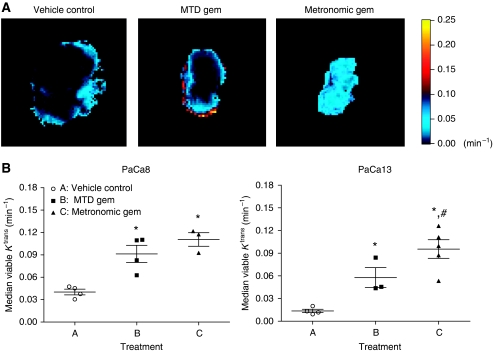
(**A**) Representative *K*^trans^ maps of PaCa13 tumours after treatment with vehicle control (0.9% saline), maximum tolerated dose gemcitabine (MTD Gem; 240 mg kg^−1^ on days 1, 8, and 15), or metronomic gemcitabine (30 mg kg^−1^, q3d) for 4 weeks. (**B**) Effects of different treatments on median values of *K*^trans^ in viable tumour tissues in PaCa8 and PaCa13 tumours. Each symbol represents an evaluable tumour from a mouse in the corresponding group. —, mean; bars, s.e. ^*^P<0.05, significantly different from that of vehicle control. ^#^*P*<0.05, significantly different from that of MTD gemcitabine.

Representative *K*^trans^ maps of PaCa13 tumours after injection of the contrast agent Gd-DTPA are shown in Figure 3A. Contrast enhancement was observed predominantly in the tumour periphery in the vehicle control and MTD gemcitabine groups. Interestingly, treatment with metronomic gemcitabine resulted in a uniformly strong contrast enhancement throughout the entire tumour. *K*^trans^ values were subsequently derived from the maps. In PaCa8 tumours, the average values for median viable *K*^trans^ were significantly higher in the metronomic and MTD gemcitabine groups (by 2.8- and 2.3-fold, respectively) compared with the vehicle control group (Figure 3B). In PaCa13 tumours, metronomic and MTD gemcitabine also significantly increased *K*^trans^ values by 6.8- and 4.1-fold, respectively, relative to the vehicle control. Moreover, average values for median viable *K*^trans^ were significantly higher in metronomic gemcitabine-treated PaCa13 tumours compared with their MTD-treated counterparts (Figure 3B).

### Tumour hypoxia, cell proliferation, and microvessel density after metronomic gemcitabine treatment

**Figure 4:**
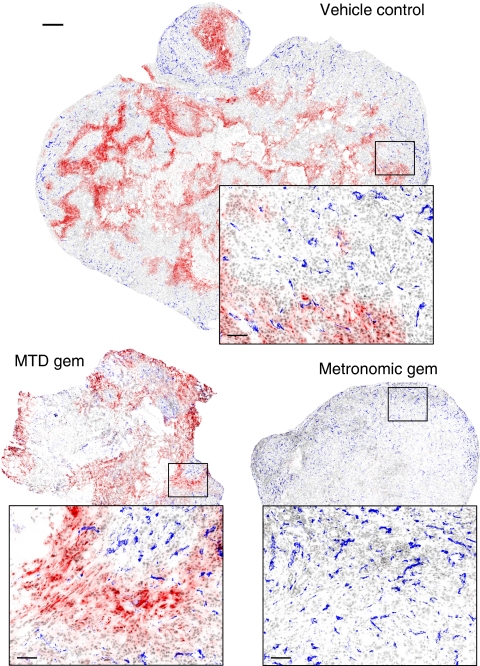
Representative composite images (scale bar: 500 *μ*m) of PaCa13 tumours showing EF5 (red), CD31 (blue), and nuclear (grey) staining after treatment with vehicle control (0.9% saline), maximum tolerated dose gemcitabine (MTD gem; 240 mg kg^−1^ on days 1, 8, and 15), or metronomic gemcitabine (30 mg kg^−1^, q3d) for 4 weeks. *Squares*, magnified images (scale bar: 83 *μ*m) of the corresponding areas in the tumour sections.

**Figure 5:**
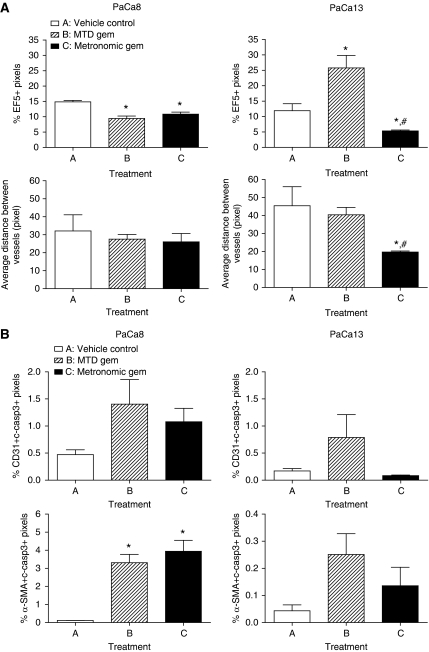
(**A**) Effects of treatment with vehicle control (0.9% saline), maximum tolerated dose gemcitabine (MTD gem; 240 mg kg^−1^ on days 1, 8, and 15), or metronomic gemcitabine (30 mg kg^−1^, q3d) for 4 weeks on hypoxia and average distance between vessels in PaCa8 and PaCa13 tumours. Columns, mean of 3–5 tumours; bars, s.e. ^*^P<0.05, significantly different from that of vehicle control. ^#^*P*<0.05, significantly different from that of MTD gemcitabine. (**B**) Effects of the three treatments on apoptosis of endothelial cells (CD31 and c-casp3 double-positive pixels) and cancer-associated fibroblasts (*α*-SMA and c-casp3 double-positive pixels) in PaCa8 and PaCa13 tumours. Columns, mean of 2–5 tumours; bars, s.e. c-casp3, cleaved-caspase 3. ^*^P<0.05, significantly different from that of vehicle control.

Figure 4 shows representative composite images of PaCa13 tumour sections from different treatment groups. Large areas of EF5+ staining (red), indicative of hypoxia, were evident in vehicle control-treated tumours. After MTD gemcitabine treatment, hypoxia remained prominent in the tumours despite their reduction in size. In sharp contrast, metronomic gemcitabine-treated tumours had little or no EF5+ staining. Quantitatively, metronomic gemcitabine decreased the percentage of EF5+ pixels by 2.2-fold in PaCa13 tumours compared with the vehicle control; MTD gemcitabine had the opposite effect, significantly increasing the percentage of EF5+ pixels by 2.2-fold (Figure 5A). In PaCa8 tumours, metronomic and MTD gemcitabine significantly and similarly reduced hypoxia (Figure 5A). Levels of tumour cell proliferation, as determined from BrdU+ staining, were similar in all treated PaCa13 tumours compared with the vehicle control. Similar observations were also evident in PaCa8 tumours (data not shown).

Microvessel density was reported as the average distance between CD31+ objects (blue staining in Figure 4) on whole tumour sections. Short and long average distances between vessels indicate high and low microvessel density, respectively. In PaCa13 tumours, only metronomic gemcitabine significantly decreased the average distance between blood vessels and therefore increased microvessel density (Figure 5A). In PaCa8 tumours, the average distance between vessels was not significantly altered by metronomic or MTD gemcitabine (Figure 5A).

### Effects of metronomic gemcitabine on endothelial cell and CAF apoptosis

None of the treatments significantly increased endothelial cell apoptosis in the two primary xenografts, as the percentages of CD31 and cleaved-caspase 3 double-positive pixels in the treated tumours were similar to those in vehicle control tumours (Figure 5B). In PaCa8 tumours, metronomic and MTD gemcitabine significantly increased CAF apoptosis by 34-fold and 29-fold, respectively, as reflected by the increased percentages of *α*SMA and cleaved-caspase 3 double-positive pixels (Figure 5B). In PaCa13 tumours, there was a trend towards increased CAF apoptosis after either gemcitabine regimen, but such increases did not reach statistical significance (Figure 5B).

### Tumour levels of growth factors and cytokines in response to metronomic gemcitabine

Metronomic gemcitabine induced significant changes in the levels of 37 and 14 out of 89 proteins in the PaCa8 and PaCa13 tumours, respectively, compared with vehicle control (Supplementary Table S1). The proteins, the levels of which were significantly reduced following metronomic gemcitabine therapy, were more numerous in PaCa8 tumours than in PaCa13 tumours (29 *vs* 13, respectively). Many of the downregulated proteins are pro-angiogenic growth factors and cytokines. In general, the decreases in the levels of these proteins caused by metronomic gemcitabine were more pronounced than those induced by MTD gemcitabine (e.g., 21-fold *vs* 6-fold for CD40 ligand, 12-fold *vs* 4.1-fold for EGF, 34-fold *vs* 20-fold for GM-CSF, 57-fold *vs* 15-fold for ICAM-1, 397-fold *vs* 75-fold for IL-1*α*, and 189-fold *vs* 18-fold for IL-8, respectively, in PaCa8 tumours; 8.9-fold *vs* 1.9-fold for VCAM-1, 2.4-fold *vs* 1.7-fold for von Willebrand factor, respectively, in PaCa13 tumours).

## Discussion

This study demonstrated that metronomic dosing of gemcitabine is active against pancreatic ductal adenocarcinoma. It is noteworthy that, although both metronomic and MTD gemcitabine induced significant tumour volume reductions in PaCa8 and PaCa13 primary xenografts, the total dose of gemcitabine administered over 4 weeks in the metronomic group was less than half of that given in the MTD group (300 *vs* 720 mg kg^−1^, respectively). Metronomic gemcitabine did not induce significant weight loss in tumour-bearing mice, indicating a lack of toxicity. These findings suggest that the use of metronomic gemcitabine in the clinic would be advantageous because such a regimen is more effective and better tolerated in terms of total drug dose than MTD weekly gemcitabine.

We then examined what might have contributed to the anti-tumour effects of metronomic gemcitabine. H&E staining of tumour sections revealed more prominent morphological changes that were characterised by more extensive mucinous differentiation, lower overall cell density, and fewer cancer cells in metronomic gemcitabine-treated tumours compared with their MTD-treated counterparts. These observations point to a direct and more pronounced effect of the metronomic regimen on pancreatic cancer cells. Indeed, it has been suggested that metronomic chemotherapy might directly affect tumour cells by inducing their differentiation (Kerbel and Kamen, 2004). Furthermore, tumour cell re-population, which often occurs during drug-free breaks between cycles of MTD chemotherapy, may also be prevented using metronomic regimens (Kerbel and Kamen, 2004).

At the functional level, our dynamic contrast-enhanced magnetic resonance imaging data demonstrated that metronomic gemcitabine treatment improves global tumour perfusion. The strong homogeneous contrast enhancement and the significant increases in *K*^trans^ values observed in metronomic gemcitabine-treated PaCa8 and PaCa13 tumours *in situ* were indicative of better tissue perfusion throughout the tumour. On the other hand, contrast enhancement was confined to the tumour periphery, and *K*^trans^ values were increased to a lesser extent in MTD gemcitabine-treated tumours. Improved tissue perfusion after metronomic gemcitabine treatment was also consistent with the substantial decreases in hypoxia observed in these tumours. This is reminiscent of the effects after vessel ‘normalisation’ as reported by Jain (2001). It is noteworthy that better tissue perfusion did not result in increased tumour cell proliferation, as BrdU staining in metronomic gemcitabine-treated tumours was not significantly different from that in vehicle control- or MTD gemcitabine-treated tumours. Improved vascular function is likely to promote the delivery of subsequent doses of gemcitabine itself to the tumour; thus, the previously reported poor tumour penetration of gemcitabine, even when administered at MTD (Huxham *et al*, 2004), might be alleviated with metronomic dosing regimens. As significant hypoxia has been detected in clinical pancreatic cancer (Koong *et al*, 2000), and hypoxia contributes to radioresistance and chemoresistance, metronomic gemcitabine-induced decreases in hypoxia could potentially enhance the efficacy of subsequently administered radiotherapy or MTD-based chemotherapy. The increases in *K*^trans^ values observed in metronomic gemcitabine-treated PaCa8 and PaCa13 tumours might seem to contradict the reductions in *K*^trans^ values that have often been reported after treatment with ‘dedicated’ anti-angiogenic agents (Morgan *et al*, 2003; Batchelor *et al*, 2007), and more recently with metronomic capecitabine plus celecoxib (Steinbild *et al*, 2007). However, the post-treatment decreases in *K*^trans^ values in these studies were not always accompanied by tumour volume reductions. It is possible that tumour volume reductions induced by metronomic gemcitabine in PaCa8 and PaCa13 tumours might lead to more substantial decreases in interstitial fluid pressure, thereby markedly improving the patency of tumour blood vessels and, in turn, tumour blood flow. As *K*^trans^ is a mixed measure of tumour blood flow and vascular permeability (Strecker *et al*, 2003; Yankeelov *et al*, 2006), an overall increase in *K*^trans^ might be detected if the increase in tumour blood flow is greater than the decrease in vascular permeability. It is also important to highlight the fact that, although metronomic and MTD regimens of gemcitabine induced similar tumour volume reductions in our studies, the two treatments resulted in different patterns of perfusion.

Given that metronomic chemotherapy has been previously associated with anti-angiogenesis (Browder *et al*, 2000; Klement *et al*, 2000; Bertolini *et al*, 2003; Hamano *et al*, 2004; Shaked *et al*, 2005), the effects of metronomic *vs* MTD gemcitabine on pancreatic tumour vasculature were investigated. PaCa13, but not PaCa8, tumours showed significant increases in microvessel density after metronomic gemcitabine treatment. MTD gemcitabine had no effect on microvessel density in both xenograft lines. Neither metronomic nor MTD gemcitabine significantly increased endothelial cell apoptosis in any of the xenografts. However, it should be emphasised that microvessel density and possibly endothelial cell apoptosis are time-dependent measures during anti-angiogenesis-based therapies (Hlatky *et al*, 2002). Any significant endothelial cell dropout might have occurred shortly after the first or second dose of drug, similar to that previously reported after cyclophosphamide treatment (Browder *et al*, 2000), and therefore might not have been captured at the end of the 4-week treatment period in our study. The anti-angiogenic and anti-tumour efficacy of metronomic gemcitabine in our primary xenograft studies could be further attributed to its ability to induce marked decreases, more so than MTD gemcitabine, in a multitude of pro-angiogenic growth factors and cytokines including EGF, IL-1*α*, IL-8, MCP-1, ICAM-1, VCAM-1, and others (Supplementary Table S1). Anti-cancer treatment regimens that cause broad-spectrum disruption of the tumour growth factor/cytokine network to a greater degree are likely to be more efficacious than those that only affect a small number of these molecules to a lesser extent. It is conceivable that the more prominent decreases in pro-angiogenic growth factors and cytokines after metronomic gemcitabine therapy ultimately improved tumour vascular function and tissue perfusion, and led to a corresponding reduction in tumour hypoxia.

Furthermore, metronomic gemcitabine significantly increased apoptosis of CAFs in PaCa8 tumours, suggesting that other stromal components could also be targets of metronomic chemotherapy regimens. In pancreatic ductal adenocarcinoma, this could be therapeutically significant, because one of the hallmarks of the malignancy is the presence of a strong desmoplastic reaction that is associated with extensive fibroblast proliferation and modified extracellular matrix deposition. A myriad of pro-angiogenic molecules, such as VEGF and EGF among others, are known to be upregulated in CAFs (Bhowmick *et al*, 2004). Increasing apoptosis in this cell population would thus deplete the supply of these molecules and, in turn, restrict angiogenesis and inhibit tumour growth more effectively. The lack of significant increases in CAF apoptosis in metronomic gemcitabine-treated PaCa13 tumours might be explained by the differential sensitivity of individual patient's CAFs to such treatment. It should be emphasised that PaCa8 and PaCa13 are primary xenografts established from surgically resected human pancreatic tumour tissues. Given the inherent heterogeneity of individual patient's tumours and their differential responses to treatments in the clinical situation, it is not surprising that metronomic and MTD gemcitabine resulted in different profiles of change of pro-angiogenic growth factors and cytokines, and, similarly, in differences in the levels of CAF apoptosis in PaCa8 and PaCa13 tumours. This highlights the value of primary human tumour models in the pre-clinical evaluation of experimental treatment modalities (Rubio-Viqueira *et al*, 2006). In addition to anti-angiogenesis, immunostimulation has also been shown to mediate the anti-tumour effects of metronomic cyclophosphamide (Hermans *et al*, 2003; Ghiringhelli *et al*, 2004, 2007; Loeffler *et al*, 2005). The effect of metronomic gemcitabine on the immune system, however, has not been reported in the literature, although a recent study showed that conventional MTD gemcitabine regimen (3 weeks on, 1 week off) reduces CD8^+^ T cells, increases CD11c^+^ dendritic cells, but does not significantly alter the number of CD4^+^ T cells in pancreatic cancer patients after one cycle of treatment when compared with pre-treatment baseline (Soeda *et al*, 2009). The possibility that immunomodulation may have a role in mediating the anti-tumour effects of metronomic gemcitabine cannot be excluded.

In conclusion, our results showed that metronomic gemcitabine is well tolerated and more effective than MTD gemcitabine in pancreatic cancer. It markedly reduces tumour levels of various pro-angiogenic molecules, and improves tumour vascular function and tissue perfusion (as reflected by the increase in *K*^trans^ and the decrease in tumour hypoxia). Pancreatic ductal adenocarcinoma is characterised by a dense fibrovascular stroma consisting of different cell types (e.g., cancer-associated fibroblasts, endothelial cells, immune cells, and so on) (Bardeesy and DePinho, 2002). These assorted populations of stromal cells and pancreatic cancer cells are unlikely to all be in a susceptible growth phase at the time when a single MTD of gemcitabine is administered once a week; it follows that more frequent dosing at a lower but still effective concentration of the drug would increase the probability of eliminating or inducing differentiation in more cells of any cell type (Kamen *et al*, 2006). Considering that suboptimal blood flow (Komar *et al*, 2009) and hypoxia (Koong *et al*, 2000) contribute to chemotherapy or radiotherapy failure in pancreatic cancer, it would be interesting to evaluate whether the tumour physiological changes (i.e., increased perfusion and decreased hypoxia) induced by metronomic gemcitabine treatment as described in this study may be exploited for the design of more effective combination therapies. Oral formulations of an increasing number of chemotherapeutic drugs are now available or are being developed. The anti-tumour efficacy of oral gemcitabine (Veltkamp *et al*, 2008), used in a metronomic regimen similar to the one described herein, warrants clinical investigation in pancreatic cancer.

## References

[bib1] Bardeesy N, DePinho RA (2002) Pancreatic cancer biology and genetics. Nat Rev Cancer 2: 897–909 12459728 10.1038/nrc949

[bib2] Batchelor TT, Sorensen AG, di Tomaso E, Zhang WT, Duda DG, Cohen KS, Kozak KR, Cahill DP, Chen PJ, Zhu M, Ancukiewicz M, Mrugala MM, Plotkin S, Drappatz J, Louis DN, Ivy P, Scadden DT, Benner T, Loeffler JS, Wen PY, Jain RK (2007) AZD2171, a pan-VEGF receptor tyrosine kinase inhibitor, normalizes tumor vasculature and alleviates edema in glioblastoma patients. Cancer Cell 11: 83–95 17222792 10.1016/j.ccr.2006.11.021 PMC2748664

[bib3] Bertolini F, Paul S, Mancuso P, Monestiroli S, Gobbi A, Shaked Y, Kerbel RS (2003) Maximum tolerable dose and low-dose metronomic chemotherapy have opposite effects on the mobilization and viability of circulating endothelial progenitor cells 1. Cancer Res 63: 4342–4346 12907602

[bib4] Bhowmick NA, Neilson EG, Moses HL (2004) Stromal fibroblasts in cancer initiation and progression. Nature 432: 332–337 15549095 10.1038/nature03096 PMC3050735

[bib5] Boven E, Schipper H, Erkelens CA, Hatty SA, Pinedo HM (1993) The influence of the schedule and the dose of gemcitabine on the anti-tumour efficacy in experimental human cancer. Br J Cancer 68: 52–56 8318420 10.1038/bjc.1993.285 PMC1968329

[bib6] Braakhuis BJ, Ruiz van Haperen VW, Boven E, Veerman G, Peters GJ (1995) Schedule-dependent antitumor effect of gemcitabine in *in vivo* model system. Semin Oncol 22: 42–46 7481844

[bib7] Braakhuis BJ, van Dongen GA, Vermorken JB, Snow GB (1991) Preclinical *in vivo* activity of 2′, 2′-difluorodeoxycytidine (Gemcitabine) against human head and neck cancer. Cancer Res 51: 211–214 1988086

[bib8] Browder T, Butterfield CE, Kraling BM, Shi B, Marshall B, O'Reilly MS, Folkman J (2000) Antiangiogenic scheduling of chemotherapy improves efficacy against experimental drug-resistant cancer. Cancer Res 60: 1878–1886 10766175

[bib9] Buckstein R, Kerbel RS, Shaked Y, Nayar R, Foden C, Turner R, Lee CR, Taylor D, Zhang L, Man S, Baruchel S, Stempak D, Bertolini F, Crump M (2006) High-dose celecoxib and metronomic ‘low-dose’ cyclophosphamide is an effective and safe therapy in patients with relapsed and refractory aggressive histology non-Hodgkin's lymphoma. Clin Cancer Res 12: 5190–5198 16951238 10.1158/1078-0432.CCR-06-0474

[bib10] Burris III HA, Moore MJ, Andersen J, Green MR, Rothenberg ML, Modiano MR, Cripps MC, Portenoy RK, Storniolo AM, Tarassoff P, Nelson R, Dorr FA, Stephens CD, Von Hoff DD (1997) Improvements in survival and clinical benefit with gemcitabine as first-line therapy for patients with advanced pancreas cancer: a randomized trial. J Clin Oncol 15: 2403–2413 9196156 10.1200/JCO.1997.15.6.2403

[bib11] Colleoni M, Orlando L, Sanna G, Rocca A, Maisonneuve P, Peruzzotti G, Ghisini R, Sandri MT, Zorzino L, Nole F, Viale G, Goldhirsch A (2006) Metronomic low-dose oral cyclophosphamide and methotrexate plus or minus thalidomide in metastatic breast cancer: antitumor activity and biological effects. Ann Oncol 17: 232–238 16322118 10.1093/annonc/mdj066

[bib12] Dellapasqua S, Bertolini F, Bagnardi V, Campagnoli E, Scarano E, Torrisi R, Shaked Y, Mancuso P, Goldhirsch A, Rocca A, Pietri E, Colleoni M (2008) Metronomic cyclophosphamide and capecitabine combined with bevacizumab in advanced breast cancer. J Clin Oncol 26(30): 4899–4905 18794539 10.1200/JCO.2008.17.4789

[bib13] Garcia AA, Hirte H, Fleming G, Yang D, Tsao-Wei DD, Roman L, Groshen S, Swenson S, Markland F, Gandara D, Scudder S, Morgan R, Chen H, Lenz HJ, Oza AM (2008) Phase II clinical trial of bevacizumab and low-dose metronomic oral cyclophosphamide in recurrent ovarian cancer: a trial of the California, Chicago, and Princess Margaret Hospital phase II consortia. J Clin Oncol 26: 76–82 18165643 10.1200/JCO.2007.12.1939

[bib14] Ghiringhelli F, Larmonier N, Schmitt E, Parcellier A, Cathelin D, Garrido C, Chauffert B, Solary E, Bonnotte B, Martin F (2004) CD4+CD25+ regulatory T cells suppress tumor immunity but are sensitive to cyclophosphamide which allows immunotherapy of established tumors to be curative. Eur J Immunol 34: 336–344 14768038 10.1002/eji.200324181

[bib15] Ghiringhelli F, Menard C, Puig PE, Ladoire S, Roux S, Martin F, Solary E, Le Cesne A, Zitvogel L, Chauffert B (2007) Metronomic cyclophosphamide regimen selectively depletes CD4+CD25+ regulatory T cells and restores T and NK effector functions in end stage cancer patients. Cancer Immunol Immunother 56: 641–648 16960692 10.1007/s00262-006-0225-8 PMC11030569

[bib16] Hamano Y, Sugimoto H, Soubasakos MA, Kieran M, Olsen BR, Lawler J, Sudhakar A, Kalluri R (2004) Thrombospondin-1 associated with tumor microenvironment contributes to low-dose cyclophosphamide-mediated endothelial cell apoptosis and tumor growth suppression. Cancer Res 64: 1570–1574 14996710 10.1158/0008-5472.can-03-3126

[bib17] Hermans IF, Chong TW, Palmowski MJ, Harris AL, Cerundolo V (2003) Synergistic effect of metronomic dosing of cyclophosphamide combined with specific antitumor immunotherapy in a murine melanoma model. Cancer Res 63: 8408–8413 14679003

[bib18] Hertel LW, Boder GB, Kroin JS, Rinzel SM, Poore GA, Todd GC, Grindey GB (1990) Evaluation of the antitumor activity of gemcitabine (2′,2′-difluoro-2′-deoxycytidine). Cancer Res 50: 4417–4422 2364394

[bib19] Hlatky L, Hahnfeldt P, Folkman J (2002) Clinical application of antiangiogenic therapy: microvessel density, what it does and doesn't tell us 1. J Natl Cancer Inst 94: 883–893 12072542 10.1093/jnci/94.12.883

[bib20] Huxham LA, Kyle AH, Baker JH, Nykilchuk LK, Minchinton AI (2004) Microregional effects of gemcitabine in HCT-116 xenografts. Cancer Res 64: 6537–6541 15374965 10.1158/0008-5472.CAN-04-0986

[bib21] Jain RK (2001) Normalizing tumor vasculature with anti-angiogenic therapy: a new paradigm for combination therapy. Nat Med 7: 987–989 11533692 10.1038/nm0901-987

[bib22] Kamen BA, Glod J, Cole PD (2006) Metronomic therapy from a pharmacologist's view. J Pediatr Hematol Oncol 28: 325–327 16794497 10.1097/00043426-200606000-00001

[bib23] Kerbel RS, Kamen BA (2004) The anti-angiogenic basis of metronomic chemotherapy. Nat Rev Cancer 4: 423–436 15170445 10.1038/nrc1369

[bib24] Kety SS (1960) Theory of blood-tissue exchange and its applications to measurements of blood flow. Meth Med Res 8: 223–227

[bib25] Kieran MW, Turner CD, Rubin JB, Chi SN, Zimmerman MA, Chordas C, Klement G, Laforme A, Gordon A, Thomas A, Neuberg D, Browder T, Folkman J (2005) A feasibility trial of antiangiogenic (metronomic) chemotherapy in pediatric patients with recurrent or progressive cancer. J Pediatr Hematol Oncol 27: 573–581 16282886 10.1097/01.mph.0000183863.10792.d4

[bib26] Klement G, Baruchel S, Rak J, Man S, Clark K, Hicklin DJ, Bohlen P, Kerbel RS (2000) Continuous low-dose therapy with vinblastine and VEGF receptor-2 antibody induces sustained tumor regression without overt toxicity. J Clin Invest 105: 15–24 10772661 10.1172/JCI8829 PMC517491

[bib27] Komar G, Kauhanen S, Liukko K, Seppanen M, Kajander S, Ovaska J, Nuutila P, Minn H (2009) Decreased blood flow with increased metabolic activity: a novel sign of pancreatic tumor aggressiveness. Clin Cancer Res 15(17): 5511–5517 19706808 10.1158/1078-0432.CCR-09-0414

[bib28] Koong AC, Mehta VK, Le QT, Fisher GA, Terris DJ, Brown JM, Bastidas AJ, Vierra M (2000) Pancreatic tumors show high levels of hypoxia. Int J Radiat Oncol Biol Phys 48: 919–922 11072146 10.1016/s0360-3016(00)00803-8

[bib29] Kyle AH, Huxham LA, Baker JH, Burston HE, Minchinton AI (2003) Tumor distribution of bromodeoxyuridine-labeled cells is strongly dose dependent. Cancer Res 63: 5707–5711 14522888

[bib30] Laquente B, Lacasa C, Ginesta MM, Casanovas O, Figueras A, Galan M, Ribas IG, Germa JR, Capella G, Vinals F (2008) Antiangiogenic effect of gemcitabine following metronomic administration in a pancreas cancer model. Mol Cancer Ther 7: 638–647 18347150 10.1158/1535-7163.MCT-07-2122

[bib31] Leach MO, Brindle KM, Evelhoch JL, Griffiths JR, Horsman MR, Jackson A, Jayson G, Judson IR, Knopp MV, Maxwell RJ, McIntyre D, Padhani AR, Price P, Rathbone R, Rustin G, Tofts PS, Tozer GM, Vennart W, Waterton JC, Williams SR, Workman P (2003) Assessment of antiangiogenic and antivascular therapeutics using MRI: recommendations for appropriate methodology for clinical trials. Br J Radiol 76(Spec No 1): S87–S91 15456718 10.1259/bjr/15917261

[bib32] Loeffler M, Kruger JA, Reisfeld RA (2005) Immunostimulatory effects of low-dose cyclophosphamide are controlled by inducible nitric oxide synthase 1. Cancer Res 65: 5027–5030 15958544 10.1158/0008-5472.CAN-05-0646

[bib33] Lord EM, Harwell L, Koch CJ (1993) Detection of hypoxic cells by monoclonal antibody recognizing 2-nitroimidazole adducts. Cancer Res 53: 5721–5726 8242628

[bib34] Lord R, Nair S, Schache A, Spicer J, Somaihah N, Khoo V, Pandha H (2007) Low dose metronomic oral cyclophosphamide for hormone resistant prostate cancer: a phase II study. J Urol 177: 2136–2140; discussion 2140 17509300 10.1016/j.juro.2007.01.143

[bib35] Lyng H, Dahle GA, Kaalhus O, Skretting A, Rofstad EK (1998) Measurement of perfusion rate in human melanoma xenografts by contrast-enhanced magnetic resonance imaging. Magn Reson Med 40: 89–98 9660558 10.1002/mrm.1910400113

[bib36] Morgan B, Thomas AL, Drevs J, Hennig J, Buchert M, Jivan A, Horsfield MA, Mross K, Ball HA, Lee L, Mietlowski W, Fuxuis S, Unger C, O'Byrne K, Henry A, Cherryman GR, Laurent D, Dugan M, Marme D, Steward WP (2003) Dynamic contrast-enhanced magnetic resonance imaging as a biomarker for the pharmacological response of PTK787/ZK 222584, an inhibitor of the vascular endothelial growth factor receptor tyrosine kinases, in patients with advanced colorectal cancer and liver metastases: results from two phase I studies. J Clin Oncol 21: 3955–3964 14517187 10.1200/JCO.2003.08.092

[bib37] Ng SS, Tsao MS, Nicklee T, Hedley DW (2001) Wortmannin inhibits pkb/akt phosphorylation and promotes gemcitabine antitumor activity in orthotopic human pancreatic cancer xenografts in immunodeficient mice. Clin Cancer Res 7: 3269–3275 11595724

[bib38] Rubio-Viqueira B, Jimeno A, Cusatis G, Zhang X, Iacobuzio-Donahue C, Karikari C, Shi C, Danenberg K, Danenberg PV, Kuramochi H, Tanaka K, Singh S, Salimi-Moosavi H, Bouraoud N, Amador ML, Altiok S, Kulesza P, Yeo C, Messersmith W, Eshleman J, Hruban RH, Maitra A, Hidalgo M (2006) An *in vivo* platform for translational drug development in pancreatic cancer. Clin Cancer Res 12: 4652–4661 16899615 10.1158/1078-0432.CCR-06-0113

[bib39] Shaked Y, Emmenegger U, Man S, Cervi D, Bertolini F, Ben David Y, Kerbel RS (2005) The optimal biological dose of metronomic chemotherapy regimens is associated with maximum antiangiogenic activity. Blood 106: 3058–3061 15998832 10.1182/blood-2005-04-1422 PMC1895327

[bib40] Soeda A, Morita-Hoshi Y, Makiyama H, Morizane C, Ueno H, Ikeda M, Okusaka T, Yamagata S, Takahashi N, Hyodo I, Takaue Y, Heike Y (2009) Regular dose of gemcitabine induces an increase CD14^+^ monocytes and CD11c^+^ dendritic cells in patients with advanced pancreatic cancer. Jpn J Clin Oncol 39: 797–806 19797418 10.1093/jjco/hyp112

[bib41] Steinbild S, Arends J, Medinger M, Haring B, Frost A, Drevs J, Unger C, Strecker R, Hennig J, Mross K (2007) Metronomic antiangiogenic therapy with capecitabine and celecoxib in advanced tumor patients – results of a phase II study. Onkologie 30: 629–635 18063875 10.1159/000110580

[bib42] Strecker R, Scheffler K, Buchert M, Mross K, Drevs J, Hennig J (2003) DCE-MRI in clinical trials: data acquisition techniques and analysis methods. Int J Clin Pharmacol Ther 41: 603–605 14692712 10.5414/cpp41603

[bib43] Tofts PS, Brix G, Buckley DL, Evelhoch JL, Henderson E, Knopp MV, Larsson HB, Lee TY, Mayr NA, Parker GJ, Port RE, Taylor J, Weisskoff RM (1999) Estimating kinetic parameters from dynamic contrast-enhanced T(1)-weighted MRI of a diffusable tracer: standardized quantities and symbols. J Magn Reson Imaging 10: 223–232 10508281 10.1002/(sici)1522-2586(199909)10:3<223::aid-jmri2>3.0.co;2-s

[bib44] Veerman G, Ruiz van Haperen VW, Vermorken JB, Noordhuis P, Braakhuis BJ, Pinedo HM, Peters GJ (1996) Antitumor activity of prolonged as compared with bolus administration of 2′,2′-difluorodeoxycytidine *in vivo* against murine colon tumors. Cancer Chemother Pharmacol 38: 335–342 8674156 10.1007/s002800050492

[bib45] Veltkamp SA, Jansen RS, Callies S, Pluim D, Visseren-Grul CM, Rosing H, Kloeker-Rhoades S, Andre VA, Beijnen JH, Slapak CA, Schellens JH (2008) Oral administration of gemcitabine in patients with refractory tumors: a clinical and pharmacologic study. Clin Cancer Res 14: 3477–3486 18519780 10.1158/1078-0432.CCR-07-4521

[bib46] Yankeelov TE, Niermann KJ, Huamani J, Kim DW, Quarles CC, Fleischer AC, Hallahan DE, Price RR, Gore JC (2006) Correlation between estimates of tumor perfusion from microbubble contrast-enhanced sonography and dynamic contrast-enhanced magnetic resonance imaging. J Ultrasound Med 25: 487–497 16567438 10.7863/jum.2006.25.4.487

[bib47] Young SD, Whissell M, Noble JC, Cano PO, Lopez PG, Germond CJ (2006) Phase II clinical trial results involving treatment with low-dose daily oral cyclophosphamide, weekly vinblastine, and rofecoxib in patients with advanced solid tumors. Clin Cancer Res 12: 3092–3098 16707607 10.1158/1078-0432.CCR-05-2255

