# Supplementary material for: Metronomic gemcitabine suppresses tumour growth, improves perfusion, and reduces hypoxia in human pancreatic ductal adenocarcinoma
Source: Br J Cancer. 2010 Jun 8;103(1):52–60. doi: 10.1038/sj.bjc.6605727 (PMC2905290; doi:10.1038/sj.bjc.6605727)
Supplement: Supplementary Table S1 [file 6605727x1.pdf]

**Table S1** Fold changes of soluble factors after maximum tolerated dose (MTD) gemcitabine (Gem) or metronomic gemcitabine treatment compared to vehicle control in the two primary pancreatic cancer xenografts.

| | PaCa8 | |
|---|---|---|
| **Soluble factors** | MTD Gem | Metronomic Gem |
| Adiponectin | ↑ 15 | -- |
| Alpha-1 antitrypsin | ↓ 17 | ↓ 32 |
| Alpha-fetoprotein | ↓ 2.0 | ↓ 1.9 |
| Alpha-2 macroglobulin | -- | ↑ 1.5 |
| Apolipoprotein H | -- | ↑ 2.0 |
| Beta-2 macroglobulin | ↓ 4.5 | ↓ 22 |
| BDNF | -- | ↓ 4.7 |
| Cancer antigen 125 | ↓ 2.4 | ↓ 11 |
| CD40 | ↓ 8.8 | ↓ 63 |
| CD40 ligand | ↓ 6.0 | ↓ 21 |
| Epidermal growth factor | ↓ 4.1 | ↓ 12 |
| Endothelin-1 | ↓ 2.4 | ↓ 4.8 |
| Fatty acid binding protein | -- | ↓ 54 |
| Factor VII | ↓ 1.9 | -- |
| Ferritin | -- | ↓ 11 |
| Fibroblast growth factor, basic | ↑ 2.9 | -- |
| G-CSF | ↓ 14 | BDL |
| GM-CSF | ↓ 20 | ↓ 34 |
| Glutathione-S-transferase | -- | ↑ 1.7 |
| ICAM-1 | ↓ 15 | ↓ 57 |
| Immunglobulin E | ↓ 4.0 | ↑ 1.5 |
| Interferon gamma | ↓ 16 | BDL |
| Interleukin-1 alpha | ↓ 75 | ↓ 397 |
| Interleukin-1 beta | ↓ 19 | ↓ 20 |
| Interleukin-1 ra | ↓ 2.8 | ↓ 35 |
| Interleukin-10 | BDL | ↓ 2.0 |
| Interleukin-12 p70 | -- | ↑ 1.7 |
| Interleukin-13 | -- | ↑ 1.6 |
| Interleukin-15 | ↓ 2.4 | -- |
| Interleukin-2 | -- | BDL |
| Interleukin-3 | BDL | BDL |
| Interleukin-4 | ↓ 2.4 | -- |
| Interleukin-5 | -- | BDL |
| Interleukin-6 | ↓ 9.7 | BDL |
| Interleukin-8 | ↓ 18 | ↓ 189 |
| Insulin | -- | ↑ 13 |
| Lipoprotein a | ↓ 2.4 | BDL |
| MCP-1 | ↓ 30 | ↓ 81 |
| MDC | ↓ 4.2 | BDL |
| MIP-1 alpha | -- | ↑ 1.5 |
| Prostatic acid phosphatase | ↓ 5.3 | ↓ 12 |
| PAPP-A | ↓ 4.0 | -- |
| Stem cell factor | ↓ 2.5 | ↓ 7.6 |
| SGOT | ↑ 2.7 | -- |

| | PaCa13 | |
|---|---|---|
| **Soluble factors** | MTD Gem | Metronomic Gem |
| Adiponectin | ↓ 4.7 | -- |
| Alpha-fetoprotein | ↑ 2.0 | -- |
| Alpha-2 macroglobulin | ↑ 4.5 | -- |
| Apolipoprotein H | ↑ 1.8 | -- |
| Beta-2 macroglobulin | ↓ 8.3 | ↓ 3.6 |
| Cancer antigen 125 | -- | ↓ 1.9 |
| Calcitonin | ↑ 3.6 | BDL |
| C-reactive protein | BDL | ↓ 4.5 |
| Eotaxin | ↑ 3.7 | -- |
| Factor VII | ↓ 4.0 | -- |
| Fibroblast growth factor, basic | ↑ 2.9 | -- |
| GM-CSF | ↑ 3.0 | -- |
| Glutathione-S-transferase | ↓ 2.4 | -- |
| ICAM-1 | ↑ 1.8 | ↓ 1.8 |
| Interferon gamma | ↑ 3.0 | -- |
| Interleukin-1 alpha | ↑ 2.4 | -- |
| Interleukin-1 ra | ↓ 2.4 | ↓ 2.4 |
| Interleukin-12 p70 | ↑ 1.7 | -- |
| Interleukin-15 | ↑ 1.9 | -- |
| Interleukin-16 | ↑ 4.1 | ↑ 2.5 |
| Interleukin-4 | ↑ 3.2 | -- |
| Interleukin-5 | ↓ 2.2 | -- |
| Leptin | ↓ 2.0 | ↓ 1.3 |
| Lipoprotein a | BDL | ↓ 2.3 |
| MCP-1 | ↑ 4.1 | BDL |
| MMP-3 | ↓ 1.7 | -- |
| Myeloperoxidase | ↑ 2.6 | -- |
| PAI-1 | ↓ 2.8 | ↓ 5.1 |
| Stem cell factor | ↑ 3.2 | -- |
| SGOT | ↑ 2.0 | -- |
| Tissue factor | ↑ 11 | -- |
| Tumor necrosis factor-alpha | ↑ 3.2 | -- |
| Tumor necrosis factor-beta | ↑ 2.3 | -- |
| Tumor necrosis factor RII | ↓ 2.1 | ↓ 3.8 |
| Thrombopoietin | ↑ 1.7 | -- |
| Thyroid stimulating hormone | ↓ 2.0 | -- |
| VCAM-1 | ↓ 1.9 | ↓ 8.9 |
| von Willebrand factor | ↓ 1.7 | ↓ 2.4 |

--, no significant change compared to vehicle control.
↑, significant increase ($P<0.05$) compared to vehicle control.
↓, significant decrease ($P<0.05$) compared to vehicle control.
BDL, below detection limit.
Note: For the complete list of 89 human antigens/soluble factors that were quantified using the HumanMAP version 1.6, refer to http://www.rulesbasedmedicine.com/services_human_antigen.asp. Only those soluble factors whose levels were significantly altered by at least one of the treatment regimens were listed above.
